# Closed subtalar dislocation with non-displaced fractures of talus and navicular: a case report and review of the literature

**DOI:** 10.4076/1757-1626-2-8793

**Published:** 2009-09-01

**Authors:** Elias Fotiadis, Christos Lyrtzis, Theodoros Svarnas, Miltos Koimtzis, Kiriaki Akritopoulou, Byron Chalidis

**Affiliations:** 1 Orthopaedic Department, General Hospital of VeriaVeria, 59100Greece; 2 Medical School, Aristotle University of ThessalonikiThessaloniki, 54124Greece; 3 Orthopaedic Department, Avenue HospitalMelbourneAustralia

## Abstract

Closed subtalar dislocations associated with talus and navicular fractures are rare injuries. We report on a case of a 43-year-old builder man with medial subtalar dislocation that was further complicated by minimally displaced talar and navicular fractures. Successful closed reduction under general anesthesia was followed by non-weight bearing and ankle immobilization with a below-knee cast for 6 ;weeks. At 3 years post-injury, the subtalar joint was stable, the foot and ankle mobility was in normal limits and the patient could still work as a builder. However, he complained for occasionally mild pain due to the development of post-traumatic arthritis in subtalar and ankle joints. Our search in literature revealed that conservative treatment of all the successfully reduced and minimally displaced subtalar fracture-dislocations has given superior results compared to surgical management. However, even in cases with no or slight fracture displacement, avascular necrosis of the talus or arthritis of the surrounding joints can compromise the final functional outcome.

## Introduction

Subtalar dislocation is a rare ankle injury. Although it can occur in any direction, medial dislocation is the most common injury pattern [1]. The lesion is usually closed [2] as a result of a high-energy injury such as fall from a height or motor vehicle accident [1]. Associated fractures may be easily overlooked and lead to disruption of the normal bone articulation, arthritis or avascular necrosis of the talus [3].

We report a case of closed subtalar dislocation with concomitant and ipsilateral talus and navicular fractures. At 3 years postoperatively, the foot scored well in terms of stability and range of motion but post-traumatic arthritis compromised the final result. We also present our results from the review of English literature regarding the incidence and the main characteristics of the injury, as well as the outcome of the applied treatment options.

The Hospital’s Scientific Research Board approved this study, which was conducted in accordance with the World Medical Association Declaration of Helsinki of 1975 as revised in 2000. The patient was informed about his participation in the study and gave informed consent.

## Case presentation

**Figure 1.**
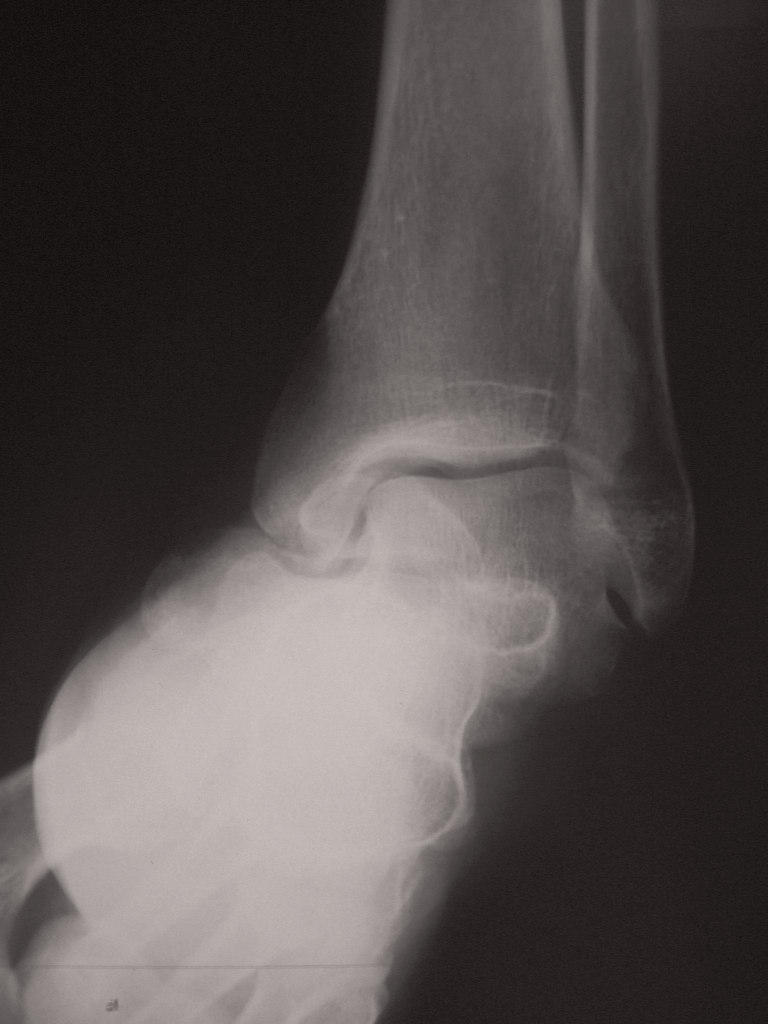

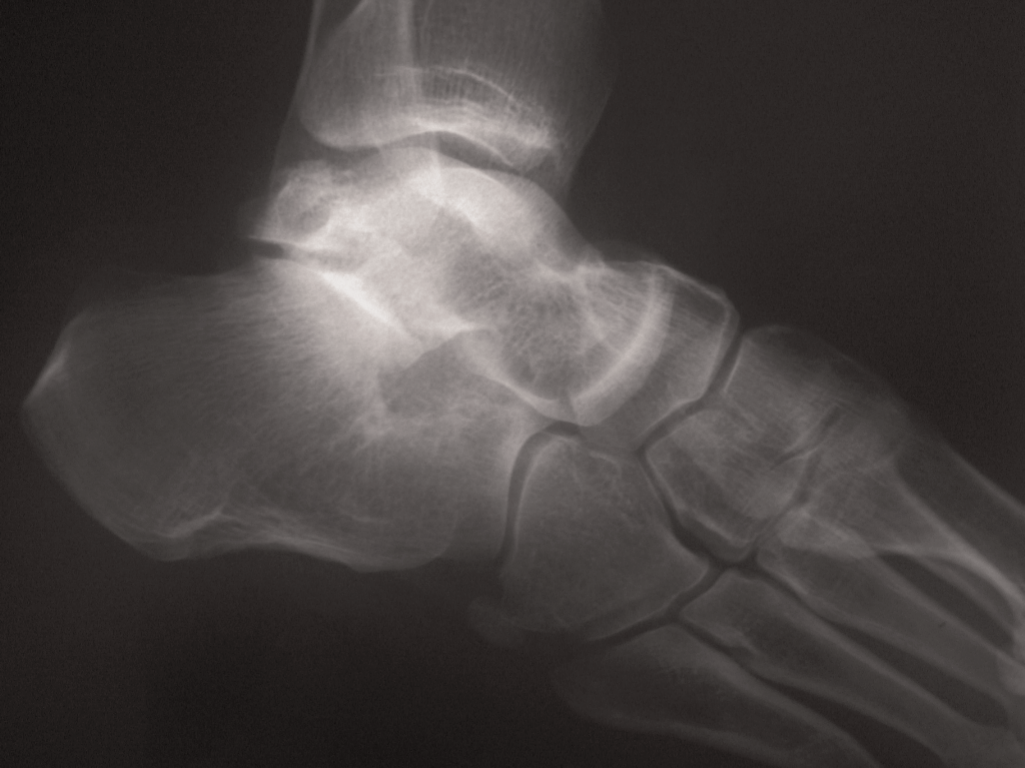
Anteroposterior **(a)** and oblique **(b)** foot radiographs illustrate medial subtalar dislocation of the right foot.

A 43-year-old Greek male builder admitted to the Accident and Emergency Department of the Hospital due to fall from a height of about 2.5 m. The patient complained of severe right ankle pain and inability to bear any weight on his extremity. In clinical examination the ankle was substantially swollen and ecchymotic, while the talonavicular and medial subtalar joints were very tender and painful to palpation. However, no neurovascular or tendon disturbances were identified. Both oblique and anteroposterior radiographs showed medial displacement of the midfoot without any evidence of bone fracture. (Figures 1a and b).

**Figure 2.**
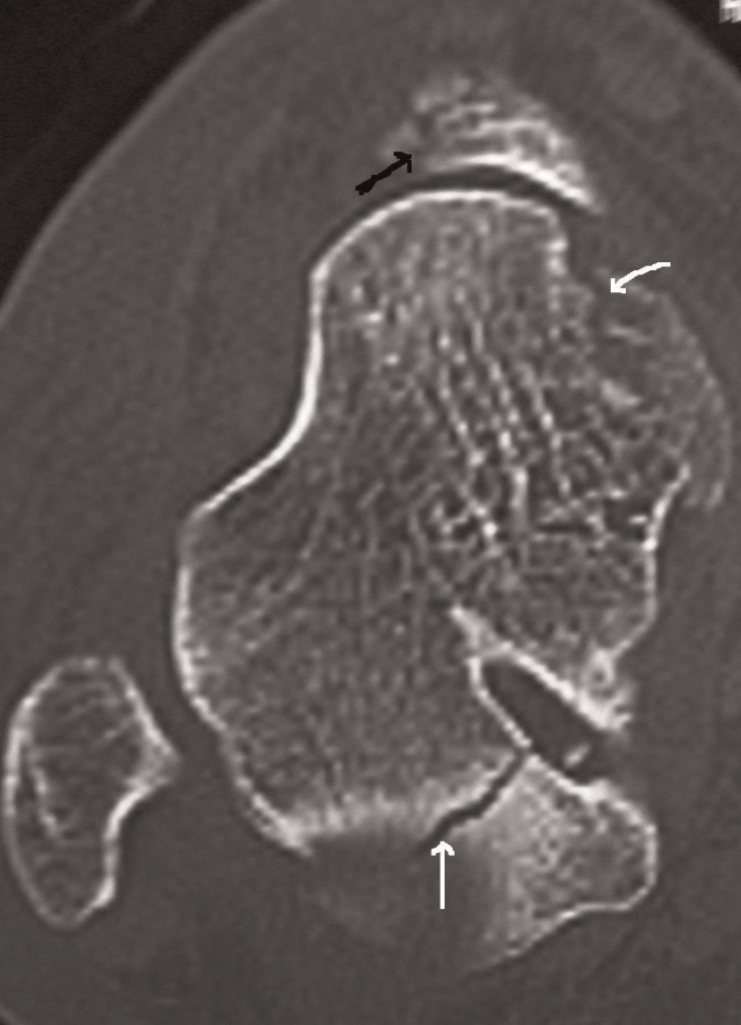
CT scan of the right foot showing two osteochondral fractures of the talus (white arrows) and an undisplaced navicular fracture (black arrow).

Under general anesthesia, the subtalar dislocation was successfully reduced with manual pressure on the head of the talus and traction, plantar flexion and pronation of the forefoot. The knee was kept flexed throughout the relocation process for eliminating the tension of the soleus muscle. Afterwards, the quality of the reduction and the stability of the subtalar joint were evaluated under fluoroscopy. As no signs of anteroposterior or mediolateral instability were recognized, the ankle was immobilized in a short leg non-weight-bearing cast for 6 weeks. A post-reduction compute tomography (CT) scan was also performed to confirm the anatomic reduction of the subtalar joint dislocation and reveal any potential fractures. The CT scan showed a nondisplaced fracture of the talus body, an osteochondral fracture of the head of the talus and a nondisplaced navicular fracture (Figure 2). Due to the benign character of all fractures, no surgical treatment was decided.

After cast removal, an intensive foot and ankle physiotherapy program was commenced for restoring the foot and ankle mobility and preventing stiffness. The patient was limited to partial weight bearing for another 2 weeks and after that time he progressed to weight bearing as tolerated.

**Figure 3.**
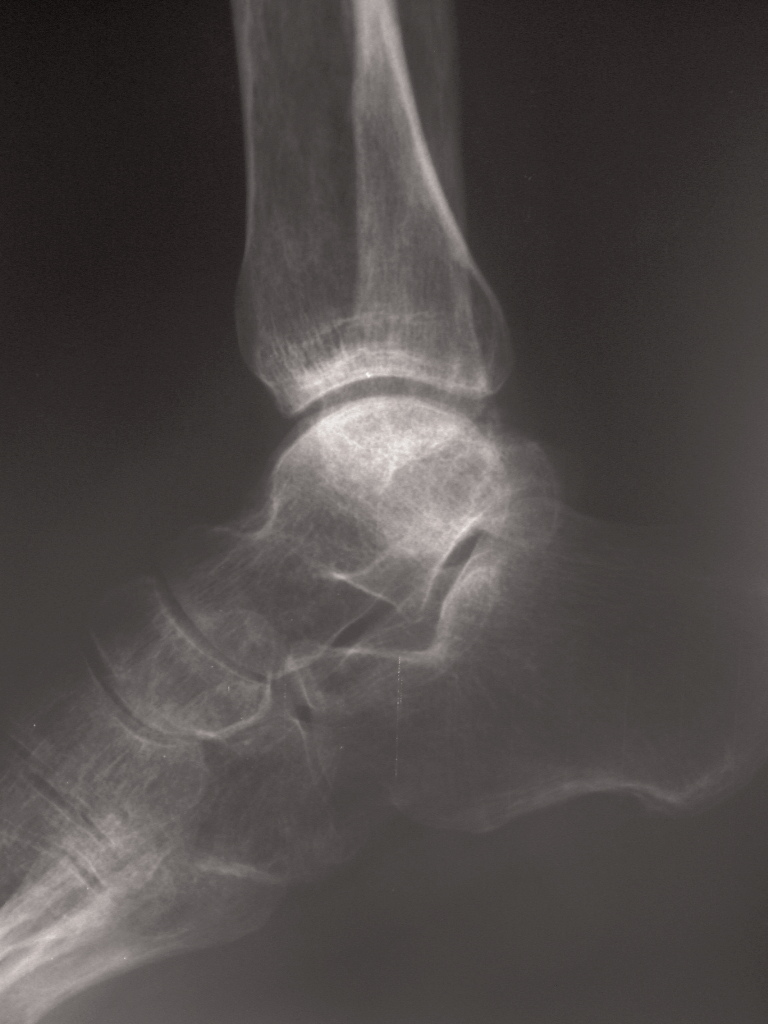
Lateral radiograph of the ankle 3 years post-injury. Sclerosis of the body of the talus and degenerative changes in ankle and subtalar joints are evident.

At 3 year follow up examination, the patient performed well in terms of foot and ankle range of motion. No signs of instability were identified. The good clinical result was also illustrated from the AOFAS [4], ankle hind foot scale, as a total score of 90 out of 100 points was achieved. Although, the patient returned to his prior to injury occupation, he complained occasionally for mild pain. The latter was attributed to the development of sclerotic changes in the body of the talus and post-traumatic osteoarthritis in subtalar and ankle joints (Figure 3).

## Discussion

Closed subtalar dislocations may be associated with concomitant intra-articular fractures of the osseous elements of foot and ankle [2]. Combined injuries can prolong the immobilization period as well as the incidence and magnitude of complications, such as arthritis of the subtalar joint or avascular necrosis of the body of talus [3].

Our search in English literature revealed 26 published studies with 328 patients suffering from closed subtalar dislocations (Table 1). In the majority of cases (86%), the lesions were treated conservatively with a below-knee cast and non-weight bearing for at least 3-6 weeks. The described results were generally good to excellent despite some residual pain or stiffness in subtalar and ankle joints [5-9]. Heppenstall et al [10] reported excellent functional results in 14 out of 19 patients after closed reduction of subtalar dislocation. However, 16 of 20 patients had significant restriction of subtalar motion and 6 of 20 ;patients had roentgenographic evidence of arthritis, after an average of 4.2 years follow-up period. Jarde et al [11] noticed good to excellent results in 24 of 35 cases with the same injury type. At the same study, 3 patients developed talar necrosis in a mean period of 1 year.

**Table 1.** Published cases of closed subtalar dislocations

Study	Year	Number of cases	Treatment	Result
Heppenstall RB et al	1980	20	A. Closed reduction (19 patients)	A. Excellent results 14, good 2, fair 2, poor 1
*J Trauma*			B. Open reduction (1 patient)	B. Poor result 1 patient
Ganel A et al	1981	3	A. Closed reduction (2 patients)	A & B. Good results
*J Foot Surg*			B. Open reduction (1 patient)	
Monson ST, Ryan JR.	1981	9	Closed reduction	A. Medial dislocation: some loss of subtalar motion
*J Bone Joint Surg (Am)*				B. Lateral dislocation: important disability
DeLee JC, Curtis R.	1982	14	A. Closed reduction (10 patients)	A. Normal ROM (5 patients)
*J Bone Joint Surg (Am)*			B. Open reduction (4 patients)	B. 50% loss of normal subtalar motion (9 patients)
Merianos P et al	1988	21	Closed reduction	A. Medial dislocations: varying degrees of disability
*Injury*				B. Lateral dislocations: serious disability
Zimmer TJ, Johnson KA.	1989	11	Closed reduction	Instability (mild- moderate): 63% of patients
*Clin Orthop Relat Res*				Restriction activity: 13% of patients
Ghrintz H et al	1989	12	Closed reduction	Dislocations without fracture: good results
*Ugeskr Laeger*				Dislocations with fracture: less favourable prognosis
Bak K, Koch JS.	1991	1	Closed reduction	Good result
*Br J Sports Med*				
Merchan EC.	1992	23	A. Closed reduction (17 patients)	A. Good results (11 patients), fair results (6 patients)
*Injury*			B. Open reduction + K-wires (6 patients)	B. Fair results (1 patient), poor result (5 patients)
Love JN et al	1995	2	A. Closed reduction (1 patient)	A & B. Mild decreased range of motion
*J Emerg Med*			B. Open reduction (1 patient)	
Ruiz Valdivieso T et al	1996	12	A. Closed reduction (10 patients)	A. Good results (6 patients), fair results (4 patients)
*Int Orthop*			B. Open reduction (2 patients)	B. Fair results (2 patients)
Jarde O et al	1996	35	A. Closed reduction (21 patients)	A. Excellent results (11 patients), Good results (10 patients)
*Rev Chir Orthop Reparatrice Appar Mot*			B. Open reduction (14 patients)	B. Good results (3 patients), fair (9 patients), poor (2 patients)
Bohay DR, Manoli A. *Foot Ankle Int*	1996	4	Closed reduction	Minimal disability and subtalar joint stiffness
Kinik H et al	1999	1	Closed reduction	Symptomless
*Int Orthop*				
Tabib W et al	2000	1	Closed reduction + K-wire	Good result
*Rev Chir Orthop Reparatrice Appar Mot*				
Kanda T et al	2001	1	Open reduction	Good result
*Foot Ankle Int*				
Perugia D et al	2002	45	Closed reduction	Good results
*Int Orthop*				
Bibbo C et al	2003	19	Closed reduction	Mean AOFAS score: 71 (fair results)
*Foot Ankle Int*				
Garofalo et al	2004	12	Closed reduction	A. Medial dislocation: excellent results (10 patients)
*J Foot Ankle Surg*				B. Lateral dislocation: fair results (2 patients)
Hadji M et al	2004	1	Closed reduction	Pain free and stable. Moderate loss of subtalar motion
*Rev Chir Orthop Reparatrice Appar Mot*				
Wagner R et al	2004	26	A. Closed reduction (20 patients)	A. Medial dislocations: Excellent results (10 patients), Good (7 patients), Fair (3 patients)
*Injury*			B. Open reduction (6 patients)	B. Lateral dislocations: Excellent results (1 patient), Good (4 patients), Poor (1 patient)
Chuo CY et al	2005	1	Open reduction	Moderate loss of subtalar motion. Mild ankle soreness
*Kaohsiung Med Sci*				
Cilli F	2006	1	Closed reduction	Excellent result
*Acta Orthop Traumatol Turc*				
Jerome JT et al	2007	1	Closed reduction	Good result
*J Foot Ankle Surg*				
Simon LC et al	2008	22	Closed reduction	Isolated dislocation: 50% excellent results
*Sportverletz Sportschaden*				Dislocation with fracture: mainly good and fair results
De Palma L et al *Arch Orthop Trauma Surg*	2008	30	Closed reduction	A. Medial dislocations: Excellent results (7 patients), good (11 patients), fair (3 patiients) B. Lateral dislocations: Good results (3 patients), fair (3 patients), poor (3 patients)

Pure dislocations seem to have a more favorable prognosis compared to combined injuries and associated fractures [12,13]. In addition, open reduction and surgical fixation of the lesion was largely related to a poor result [14]. Merchan [15], described less favorable results in almost half of the 23 patients with closed subtalar dislocation. Interestingly, 6 out of 23 patients that were treated with open reduction and K-wires fixation had fair or poor final outcome. On the other hand, Kanda et al [16] and Chuo et al [17] reported good results and only mild ankle soreness after open reduction of the dislocation. Finally, Ganel et al [18] and Love et al [19] found that conservative and surgical treatment of closed subtalar dislocations were equal in terms of ankle and foot function.

According to the published studies, there is no general agreement regarding the proper immobilization period after successful reduction of the subtalar dislocation. DeLee and Curtis [20], found that in isolated cases without concomitant fractures, 3 weeks of immobilization could offer adequate joint stability and almost normal ROM. On the contrary, there was a decrease of 50% in subtalar motion when a concomitant foot or ankle fracture existed and the immobilization period prolonged to more than 6 weeks. Similarly, Bohay and Manoli [21], stated that the factors resulting in a poor outcome after a subtalar dislocation were open lesions, bone fractures and prolonged immobilization. However, Zimmer and Johnson [22] advocated that subtalar instability (symptomatic) could occur in younger patients (average age 26 years) that treated with shorter periods of immobilization. Specifically, mild to moderate instability was developed in 62.5% of cases after a mean immobilization period of 4.4 weeks (range 3-9 ;weeks). Despite the diversity of the available clinical results, it seems that ankle immobilization should not be less than 6-8 weeks in case of associated undisplaced talus or navicular fractures [23].

The direction of dislocation seems to play also a significant role in the final functional outcome. Medial subtalar dislocations usually have shown good results when treated conservatively, while lateral dislocations have been associated with important disability [24-28]. However, Perugia et al [29] reported no significant difference in the AOFAS score between medial and lateral subtalar dislocations in a series of 45 patients. The authors pointed out that if pure low-energy subtalar dislocations were promptly reduced and immobilized for 4 weeks, a favorable outcome should be anticipated.

In the current case report, we emphasize that even careful scrutinize of the initial radiographs could not be always adequate for identifying any associated fractures. In this case, the clinical result may be complicated by stiffness and painful deformity. Therefore, we advocate further examination with CT scan after reduction of the dislocation. However, and despite the meticulous evaluation of the injured area, the current treatment methods cannot preclude the possibility of avascular necrosis of the talus and post-traumatic arthritis. These findings, which were also evident in our case, underline the severity of the injury and the magnitude of damage in both bone and soft tissue structures.

In conclusion, additional radiologic examination may be of clear benefit in all the subtalar dislocations. Conservative treatment remains the optimal treatment choice for the all the dislocation types without concomitant displaced fractures. However, the long-term performance of the foot is unpredictable due to the risks of avascular necrosis of the talus and degenerative arthritis.
